# Homogalacturonan Accumulation in Cell Walls of the Green Alga *Zygnema* sp. (Charophyta) Increases Desiccation Resistance

**DOI:** 10.3389/fpls.2019.00540

**Published:** 2019-04-25

**Authors:** Klaus Herburger, Anzhou Xin, Andreas Holzinger

**Affiliations:** ^1^ The Edinburgh Cell Wall Group, Institute of Molecular Plant Sciences, School of Biological Sciences, The University of Edinburgh, Edinburgh, United Kingdom; ^2^ Functional Plant Biology, Department of Botany, University of Innsbruck, Innsbruck, Austria

**Keywords:** cell wall, desiccation, green algae, homogalacturonan, pectin, photosynthesis, terrestrialization, Zygnematophyceae

## Abstract

Land plants inherited several traits from their green algal ancestors (Zygnematophyceae), including a polysaccharide-rich cell wall, which is a prerequisite for terrestrial survival. A major component of both land plant and Zygnematophyceaen cell walls is the pectin homogalacturonan (HG), and its high water holding capacity may have helped algae to colonize terrestrial habitats, characterized by water scarcity. To test this, HG was removed from the cell walls of *Zygnema* filaments by pectate lyase (PL), and their effective quantum yield of photosystem II (YII) as a proxy for photosynthetic performance was measured in response to desiccation stress by pulse amplitude modulation (PAM). Old filaments were found to contain more HG and are more resistant against desiccation stress but relatively lose more desiccation resistance after HG removal than young filaments. After rehydration, the photosynthetic performance recovered less efficiently in filaments with a HG content reduced by PL, independently of filament age. Immunolabeling showed that partial or un-methylesterified HG occurs throughout the longitudinal cell walls of both young and old filaments, while no labeling signal occurred when filaments were treated with PL prior labeling. This confirmed that most HG can be removed from the cell walls by PL. The initial labeling pattern was restored after ~3 days. A different form of methylesterified HG was restricted to cell poles and cross cell walls. In conclusion, it was shown that the accumulation of HG in *Zygnema* filaments increases their resistance against desiccation stress. This trait might have played an important role during the colonization of land by Zygnematophyceae, which founded the evolution of all land plants.

## Introduction

The earliest land plant fossils date to the Mid-Ordovician (Steemans et al., 2009), and it was concluded that the algal ancestors of land plants started colonizing terrestrial habits at least ~450 Myr ago (Kenrick et al., 2012). On the other hand, analyses of oxygen and carbon isotope data indicate a first expansion of terrestrial photosynthesizing organisms long before the earliest fossils, ~850 Myr ago (Knauth and Kennedy, 2009). Indeed, molecular clock estimates suggest that about 700 Myr years ago, terrestrial habitats were characterized by a community of phototrophs including bryophytes (Heckman et al., 2001). Zygnematophyceae, members of the charophyte green algae (CGA), are considered the sole ancestors of land plants (Wickett et al., 2014) and therefore must have evolved strategies to survive under terrestrial conditions. Still today, Zygnematophyceae—including their species richest genus *Zygnema*—occur worldwide in terrestrial habitats, where they are frequently exposed to abiotic stress such as low water availability (Holzinger and Karsten, 2013). In order to maintain a metabolically active state and long-term population survival under harsh terrestrial conditions (Pichrtová et al., 2016a), these algae require survival strategies at the cellular level. One such key element for stress survival might be the algal cell wall and in a broader sense the whole extracellular matrix (ECM); Zygnematophyceae lack protective covering tissues typical for their land plant descendants (e.g., epidermis), which makes the cell wall the only barrier between the algal protoplasts and the environment. In the past decades, considerable effort has been made to investigate the chemical composition of CGA’s cell walls (Domozych et al., 1980; O’Rourke et al., 2015). In particular, specific cell wall probes, such as antibodies, proved to be a powerful and time efficient tool to study the polysaccharide composition of algal cell walls at different developmental stages, under stress conditions, or in a phylogenetic context (Sørensen et al., 2011; Domozych et al., 2014; Andosch et al., 2015; Herburger and Holzinger, 2015). Together with biochemical, genomic, and transcriptomic data (Wodniok et al., 2011; Timme et al., 2012; Mikkelsen et al., 2014; Rippin et al., 2017; De Vries et al., 2018; Jensen et al., 2018), it was concluded that the most important core cell wall components of land plants—including cellulose, homogalacturonan (HG), xyloglucan, mannans, and xylans—were already present in some streptophyte green algae, and that a polysaccharide rich cell wall can be considered a prerequisite for terrestrial survival (Harholt et al., 2016). In their natural habitats, vegetative cells of *Zygnema* filaments form specialized resistant cells termed “pre-akinets” (McLean and Pessoney, 1971; Pichrtová et al., 2014a), which differ from young cells in their accumulation of starch and lipid bodies rich in C18 fatty acids (Pichrtová et al., 2016b), a reduction of chloroplast size and cell diameter and an increased resistance against osmotic and desiccation stress (Kaplan et al., 2013; Pichrtová et al., 2014a; Herburger et al., 2015). Furthermore, pre-akinete formation involves a considerable increase of the cell wall diameter, which can be attributed to an increase of pectic material in the cell wall (Herburger et al., 2018). Biochemical and immunological evidence showed that the major pectic component of late diverged CGA is HG, while other pectin-domains such as arabinans or rhamnogalacturonan I are less abundant (Sørensen et al., 2011; O’Rourke et al., 2015). HG, which is still an important component of land plant cell walls, is a linear homopolymer consisting of α-1,4-linked d-galacturonic acid (GalA) and synthesized in a highly methylesterified form (methylation at C6 carboxyl of GalA); upon secretion into the cell wall, the ester-bonds are enzymatically broken by apoplastic pectin methylesterase (Wolf et al., 2009). Thus, as shown by immunological studies, HG predominates in its de-methylesterified form in both land plant and algal cell walls (Harholt et al., 2010; Sørensen et al., 2011; Domozych et al., 2014). *In muro*, the free carboxy groups of adjacent HG monomers form complexes with Ca^2+^ ions (“egg box model”; Morris et al., 1982). These hydrated HG–Ca^2+^ complexes have a gelatinous structure and are considered to contribute to cell wall stiffness and cell wall pore size regulation (Wolf and Greiner, 2012). *In vitro* data indicate that the degree of methylesterification (DE) influences the water holding capacity of HG, where a lower DE (i.e., more free carboxy groups) might increase HG’s water holding capacity (Willats et al., 2001). This is interesting in the context of preliminary data, suggesting that water scarcity increases the thickness of pectic cell wall layers in *Zygnema irregulare* and that this is accompanied by increasing moisture content in the filaments (Fuller, 2013). Stopping experimental water scarcity decreased the diameter of the pectic layers formed during desiccation (Fuller, 2013). This suggests that the higher pectin content in the walls of old *Zygnema* cells (“pre-akinetes”), which might also contribute to the gelatinous appearance of natural *Zygnema* populations (Ettl and Gärtner, 1995), is an adaptation to low water availability by withholding moisture in the filaments. To test this hypothesis, we used the enzyme pectate lyase to remove HG from the cell walls of *Zygnema* filaments and measured their photosynthetic performance in response to desiccation stress in comparison to untreated algae. Using filaments from *Zygnema* cultures of different age (1 and 12 months) allowed us to study filaments with different pectin contents. Immunolabeling was used to visualize HG in the algal cell walls.

## Materials and Methods

### Algal Source and Material

*Zygnema circumcarinatum* (“Saalach,” SAG 2419) was grown in liquid media or on 1.5% agar plates prepared with Bold’s Basal Medium (BBM; Bischoff and Bold, 1963) for up to 12 months. Culture conditions were described in detail previously (Herburger et al., 2015). Pectate lyase (PL; E-PLYCJ, EC 4.2.2.2; from *Cellvibrio japonicus*) and endo-polygalacturonase (EPG; E-PGALUSP, EC 3.2.1.15; from *Aspergillus aculeatus*) were purchased from Megazyme Inc. (Wicklow, Ireland), the monoclonal Antibodies (mAbs) JIM5 and JIM7 from Plant Probes (Leeds, United Kingdom); other chemicals are mainly from Sigma-Aldrich (Steinheim, Germany).

### Immunocytochemistry and Histochemical Staining

Immunolabeling with the mAbs JIM5, which recognizes partially methylesterified HG (Knox et al., 1990), and JIM7 (recognizing partially but not unesterified HG; Clausen et al., 2003), was done according to Herburger et al. (2018). Briefly, 1- or 12-month-old *Zygnema* filaments were fixed (2% paraformaldehyde, 1 h), blocked with 1% BSA (30 min), and incubated with JIM5 or JIM7 (1:20 in PBS, 2 h). After rinsing, filaments were blocked again (0.5% BSA, 30 min) and incubated in the secondary antibody (FITC goat anti-rat-IgG (whole molecule) (Sigma-Aldrich); 1:100 in PBS, 2 h). After washing, fluorescence was visualized with a Zeiss Pascal 5 confocal laser-scanning microscope on a Zeiss Axiovert 200 M microscope (EX 488 nm, EM 505–550 nm, false colored green and 560 nm long pass, false colored red). Images were taken with an Axiocam MRc5 camera and z-stacks generated by merging up to 40 optical sections through a filament. Corresponding bright-field images were collected in a third channel and merged with the false color red image. As a control, the primary antibody was omitted or heat-inactivated prior to use. To remove homogalacturonan from algal cell walls, some filaments were treated with PL (3 U ml^−1^ BBM, pH 6.9) in dark and under gentle shaking for up to 24 h. Treated filaments were then subjected to immunolabeling with JIM5. Furthermore, the re-formation of the HG matrix in the cell walls was monitored during different stages of the desiccation experiment by JIM5 immunolabeling. For pectin detection, untreated and PL-treated filaments from 1- or 12-month-old *Zygnema* cultures were incubated with 0.02% (w/v) ruthenium red in BBM for 20 min, and red color was visualized with a Zeiss Axiovert 200 M microscope (Chamberlain, 1933).

### Cryofixation and Immunogold Labeling

Algal filaments were cryo-fixed and freeze-substituted in a Leica EMPACT high-pressure freezer (Leica Microsystems) and a Leica EM AFS (Lütz-Meindl and Aichinger, 2004), followed by embedding in LR-White (London Resin Company Ltd.). Ultrathin sections were prepared with a Leica ultramicrotome and immunogold labeling with JIM7, and control experiments were done according to Holzinger et al. (2000) with modifications. Briefly, ultrathin sections were blocked, incubated with JIM 7, washed, and stained with a 10 nm gold conjugated anti rat IgA secondary AB (Sigma-Aldrich). After rinsing, samples were investigated using a Zeiss Libra 120 transmission electron microscope (80 kV) connected to a ProScan 2 k SSCCD camera, controlled with OSIS iTEM software.

### Photosynthetic Performance During Desiccation and After Rehydration

**Figure 1:**
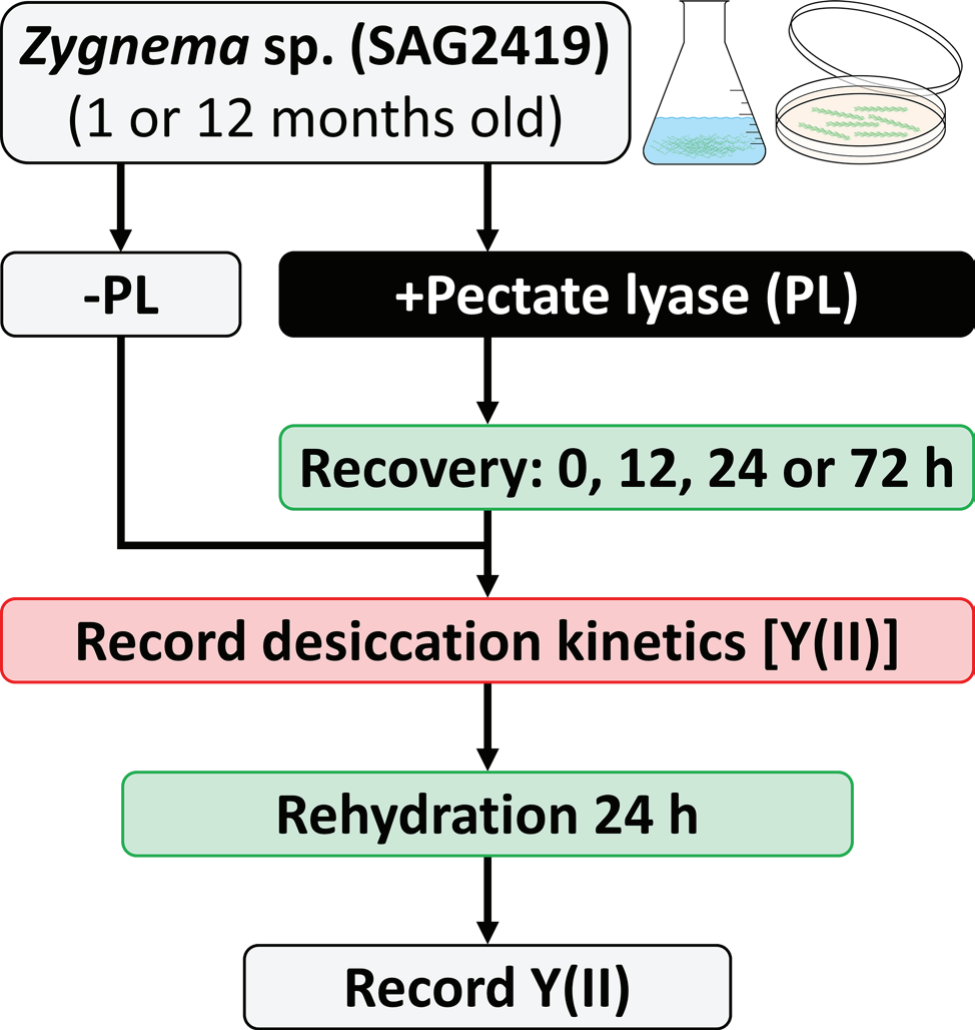
Experimental setup for enzymatic homogalacturonan removal and measuring the photosynthetic performance in response to desiccation treatment. Algal filaments are desiccated on glass fiber filters in a desiccation chamber.

The consequence of desiccation followed by rehydration on the effective quantum yield of PSII [Y(II)] in *Zygnema* was determined according to Herburger et al. (2015). Desiccation kinetics were recorded for (1) untreated samples and (2) samples treated with PL (3 units ml^−1^ BBM, pH 6.9, up to 24 h), which were either immediately subjected to desiccation treatment or after a recovering period of 12, 24, or 72 h (Figure 1 summarizes the experimental setup). For desiccation, filaments were transferred onto GF/F glass fiber filters (Whatman, Dassel, Germany) previously soaked with BBM (~220 μl), placed in a desiccation chamber (Karsten et al., 2014) and dried at ~84% relative air humidity (RH; set with KCl; Greenspan, 1977) and ~40 μmol photons m^−2^ s^−1^ for up to 10 h (*n* = 6). Y(II) was determined continuously with a pulse-amplitude modulated fluorimeter (PAM 2500; Heinz Walz GmbH, Effeltrich, Germany). After 10 h or after Y(II) reached 0, filters were rehydrated with BBM for 24 h, placed in a fresh chamber filled with water, and the Y(II) was measured again.

### Comparing Homogalacturonan Content in Young and Old Zygnema Cell Walls

From 1- and 12-month-old *Zygnema* cultures, the alcohol insoluble residue (AIR) was prepared (Foster et al., 2010); briefly, ~0.5 g fresh weight of *Zygnema* filaments were 70% ethanol extracted for 2 h, pelleted, and re-extracted overnight in the same solution until the supernatant was transparent. Then, the pellet was suspended in pure acetone and air dried. 5 mg of AIR were digested with 3 U/ml PL (in 200 μl 50 mM collidine buffer, pH 8.0) at 25°C for 3 days. After reaction stop, the supernatant was dried and re-dissolved in 20 μl EtOH, and digestion products were separated by thin-layer chromatography (TLC). In an additional experiment, 0.4 mg of young or old *Zygnema* AIR was hydrolyzed with 2 M TFA (120°C, 1 h), dried, re-dissolved in 20 μl EtOH, and analyzed by TLC. Furthermore, 1 mg of old *Zygnema* AIR was digested with 10 U/ml EPG [in 100 μl pyridine/acetic acid/water (1/1/98, v/v/v), pH 4.5] at 25°C for 3 days and then dried, re-dissolved in 20 μl EtOH, and subjected to TLC. Control groups lacked enzyme or TFA. TLC plates were developed in butan-1-ol:acetic acid:water (2:1:1; BAW; two ascents) on 60 F_254_ silica-gel plates (Merck, Darmstadt, Germany). Sugar bands were stained with thymol/H_2_SO_4_ (Jork et al., 1994) and quantified using ImageJ. Loaded markers: rhamnose (Rha), xylose (Xyl), mannose (Man), arabinose (Ara), glucose (Glc), galactose (Gal), glucuronic acid (GlcA), galacturonic acid (GalA), and PL products, GalA_1–3_–4,5-unsaturated 4-deoxyuronic acid (ΔUA) oligosaccharides.

## Results

### Pectate Lyase Removes Homogalacturonan Detectable by Immunolabeling

**Figure 2:**
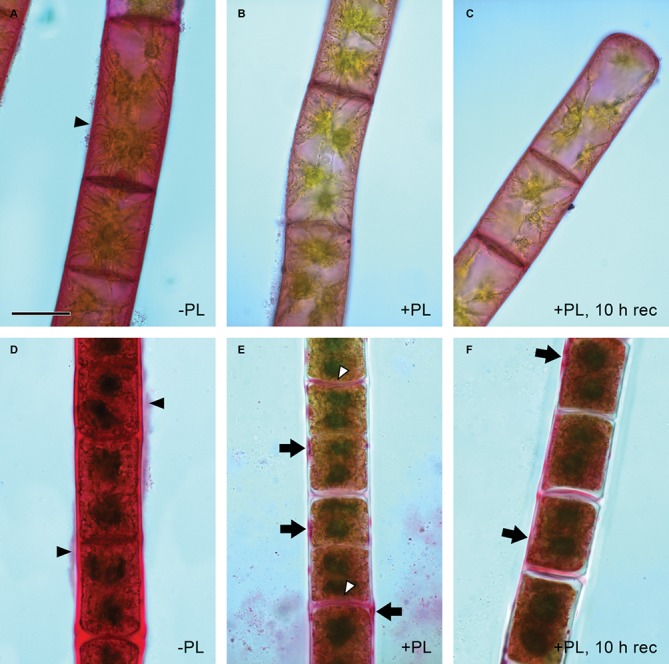
Detection of pectin by ruthenium red staining. Young **(A–C)** and old **(D,E)** filaments. **(A,D)** No pectate lyase treatment, **(B,E)** PL treatment and **(C,F)** PL treatment followed by 10 h recovery. **(A,D)** Red staining in cell walls and pectic sheath layer (arrowheads), **(E)** patchy staining in outer cell walls (arrows) and throughout most cross cell walls (arrowheads), **(F)** patchy staining in **(E)** replaced by more continuous staining (arrows). Scale bar = 20 μm.

**Figure 3:**
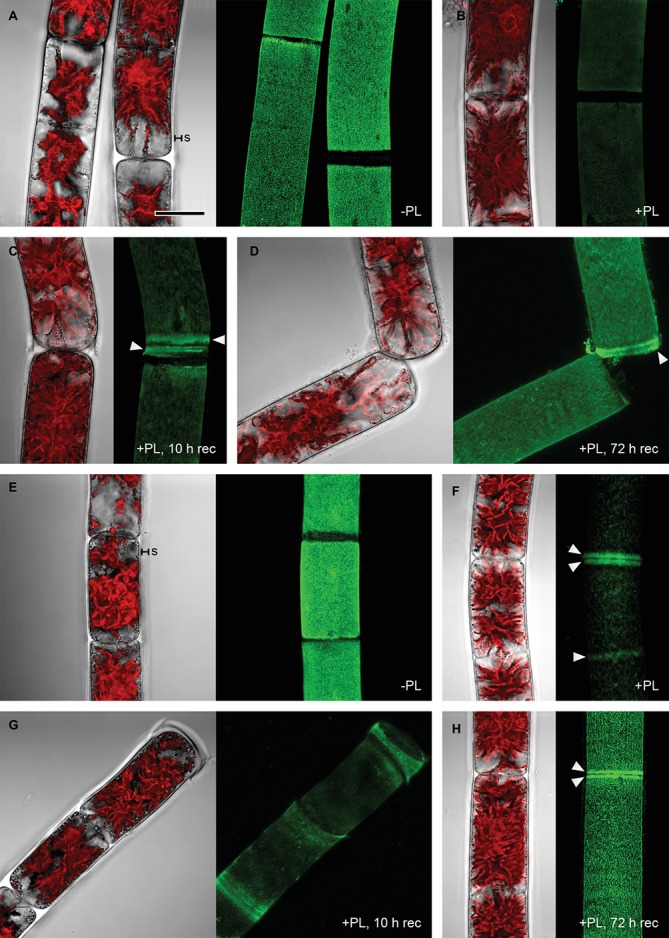
Detection of partially methylesterified homogalacturonan by mAb JIM5 in *Zygnema* filaments. Young **(A–D)** and old **(E–H)** filaments were JIM5-labeled (green, z-stacks) before pectate lyase treatment (−PL), after PL treatment (+PL) or after PL treatment followed by a recovery period (+PL, 10–12 h rec). The corresponding bright-field images include red chloroplast autofluorescence. **(A,E)** pectic sheath layer (s), **(B,F)** Labeling strongest close to cross cell walls (arrowheads), **(C,G)** re-formation of HG matrix next to cross cell walls (arrowheads), **(D,H)** labeling strongest in cross cell wall areas (arrowheads). Scale bar = 20 μm.

Pectate lyase catalyzes the endolytic and exolytic cleavage of polygalacturonic acid by β-elimination (Herron et al., 2000) and was therefore used to remove demethylesterified HG from *Zygnema* cell walls. Ruthenium red, which is commonly used to visualize pectins, stained both longitudinal and cross cell walls of filaments from 1-month-old cultures red (Figure 2A). Filaments treated with PL (Figure 2B), and recovering for 10 h after PL treatment (Figure 2C) showed weaker staining. Filaments from 12-month-old cultures showed strong red staining in all cell wall areas and in amorphous sheaths surrounding the filaments (Figure 2D), the latter being less visible in young filaments (Figure 2A). PL treatment of old filaments resulted in a patchy labeling pattern in longitudinal but not cross cell walls (Figure 2E). A 10 h recovering period resulted in a slightly stronger staining in outer cell walls (Figure 2F). Labeling with the mAb JIM5 showed that HG epitopes were abundant across the longitudinal cell walls of filaments from 1 month cultures before PL treatment. In contrast to Ruthenium red staining, signal was lacking in cross cell walls between individual cells and the pectic sheath layer (Figure 3A). PL treatment prior immunolabeling strongly and uniformly reduced the JIM5 signal in the cell walls (Figure 3B). A recovery period of 10 h after PL treatment only partly restored the mAb signal with a maximum in band shaped areas adjacent to cross cell walls (Figure 3C). In contrast, 72 h of recovery restored the initial signal (Figure 3D). In filaments from 12-month-old cultures (Figures 3E,F), the JIM5 labeling pattern was similar: Untreated filaments showed strong signal in longitudinal but not cross cell walls or the pectic sheath layer (Figure 3E). However, filaments treated with PL showed signal in band-shaped areas close to cross cell walls, while a patchy labeling pattern occurred in the rest of the longitudinal cell walls (Figure 3F). Ten hours of recovery after PL treatment restored the mAb signal partly and reduced the patchy labeling pattern (Figure 3G). Filaments recovering for 72 h showed full JIM5 signal restoration (Figure 3H).

### Another Form of Partially Methylesterified Homogalacturonan Localizes at Cell Poles and Cross Cell Walls

**Figure 4:**
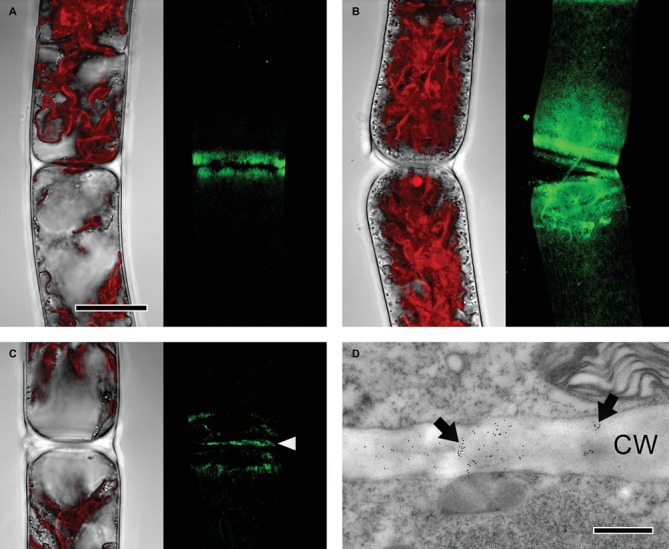
Detection of partially methylesterified homogalacturonan by JIM7 in *Zygnema* filaments. JIM7 labeling was visualized in young **(A,C,D)** and old **(B)** filaments by confocal laser scanning microscopy (**A–C**; green, z-stacks) and TEM **(D)**. Corresponding bright-field images in **(A–C)** include red chloroplast autofluorescence. **(A,B)** Labeling strongest in areas close to cross cell walls, **(C)** signal in cross cell wall (arrowhead), **(D)** immunogold-labeling in cross cell wall (arrows; CW). Scale bars = 20 μm **(A–C)**, 500 nm **(D)**.

JIM7, recognizing partially but not unesterified HG, bound to cell wall areas close to cell poles in both young and old *Zygnema* filaments (Figures 4A,B). Labeling was stronger in old filaments, whereas the signal decreased gradually toward the center of the cells (Figure 4B). Furthermore, labeling occurred in cross cell walls (Figures 4C,D).

### Removal of Homogalacturonan Reduces Resistance Against Desiccation Stress

**Figure 5:**
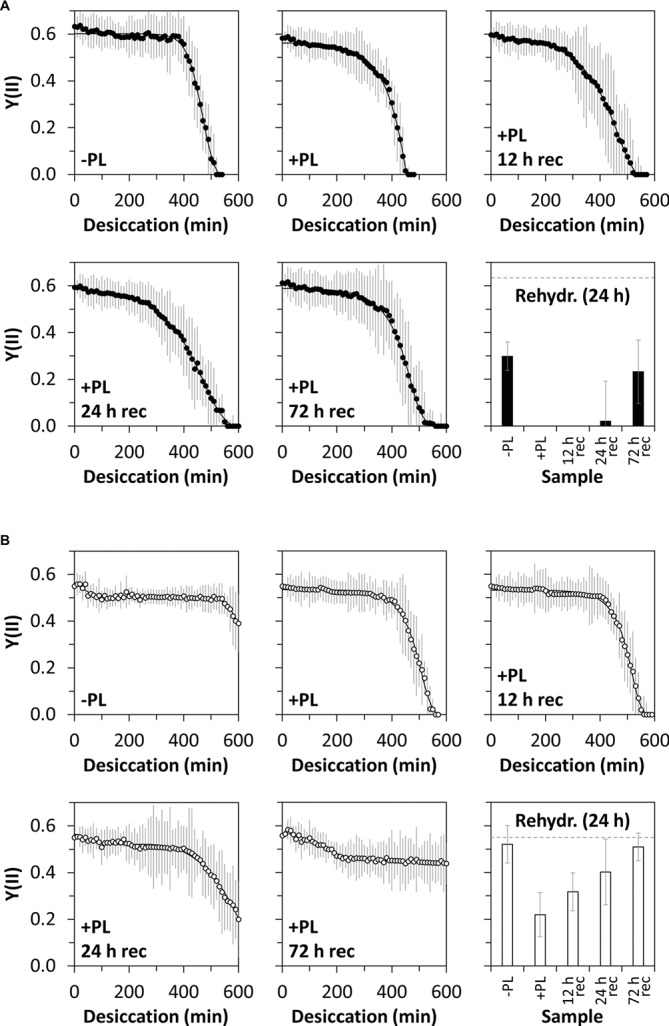
Effective quantum yield of PSII [Y(II)] in response to desiccation and pectate lyase (PL) treatment. Young **(A)** or old **(B)** algal filaments were desiccated at ~84% RH for up to 10 h after no PL treatment (−PL), after PL treatment (+PL) or after PL treatment followed by 12, 24, or 72 h recovery (+PL, 12 h rec–72 h rec). After recording desiccation kinetics, all samples were rehydrated for 24 h and their Y(II) measured. The dashed grey lines on the rehydration charts indicate the initial Y(II) of −PL samples. *n* = 6 ± SD.

The desiccation kinetics of untreated algal filaments and filaments treated with PL to remove homogalactuornan from the cell wall were compared to evaluate the role of HG in desiccation resistance. Algae from 1-month-old cultures and desiccated at 84% RH maintained their initial Y(II) (0.63) for ~6.5 h, before it dropped linearly to 0 within ~2 h (Figure 5A). The initial Y(II) of PL-treated algae was maintained for a shorter period (~5 h) and reached 0 after ~7.5 h of desiccation. Filaments, which were recovered after PL treatment for up to 72 h, showed similar desiccation kinetics; however, a Y(II) above 0 was maintained longer when compared with samples lacking recovery after PL treatment (Figure 5A). Desiccated filaments, which were not treated with PL, recovered their Y(II) partially after a 24 h rehydration period (~50% of the initial value; Figure 5A). Only PL treated samples, which were recovered for 72 h after desiccation stress, recovered their Y(II) to ~50% of the initial value (Figure 5A). The Y(II) of untreated algae from 12-month-old cultures did not reach 0 after 10 h of desiccation at 84% RH (Figure 5B). In contrast, PL treatment of 12 month-old samples caused a linear decline of the initial Y(II) (0.55) after ~7 h and Y(II) reached 0 after ~9.5 h. A 12 h recovering time between PL treatment and desiccation treatment produced similar results. However, longer recovering periods (24 or 72 h) prevented the Y(II) to drop to 0 even after 10 h of desiccation (Figure 5B). Rehydrating desiccated algae for 24 h allowed all samples (−PL, +PL, or +PL followed by recovering periods up to 72 h) to recover their Y(II), while untreated samples and samples recovered for 72 h after PL treatment restored their initial Y(II) (Figure 5B).

### Higher Amount Gala in Old Zygnema AIR After TFA Hydrolysis

**Figure 6:**
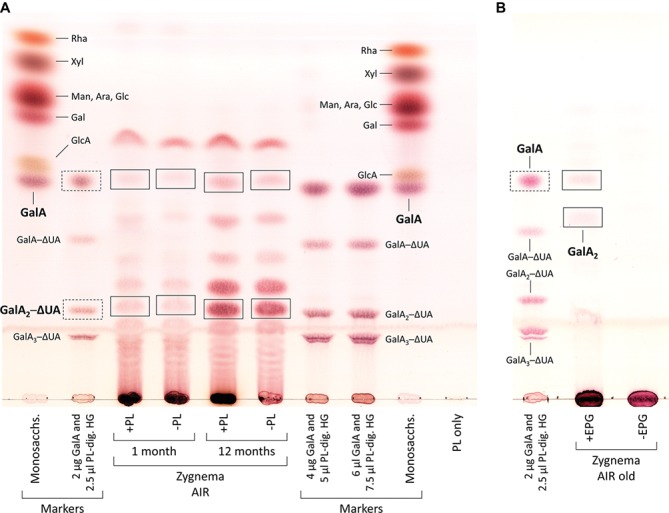
Enzymatic release of HG-derived oligosaccharides from cell walls of young and old *Zygnema* filaments. **(A)** AIR samples were either treated with PL (+PL) or buffer only (−PL, controls) and digestion products separated by thin-layer chromatography (TLC). **(B)** Old Zygnema AIR treated with EPG (+EPG) or buffer (−EPG) and product separation by TLC. Monosaccharide and galacturonic acid oligosaccharides (GalA_(1–3)_–ΔUA, as concentration gradient) markers are shown in separate tracks. Expected HG oligosaccharide and GalA bands in *Zygnema* AIR (rectangles) and GalA_1–3_–ΔUA marker bands (rectangles with dashed lines).

**Figure 7:**
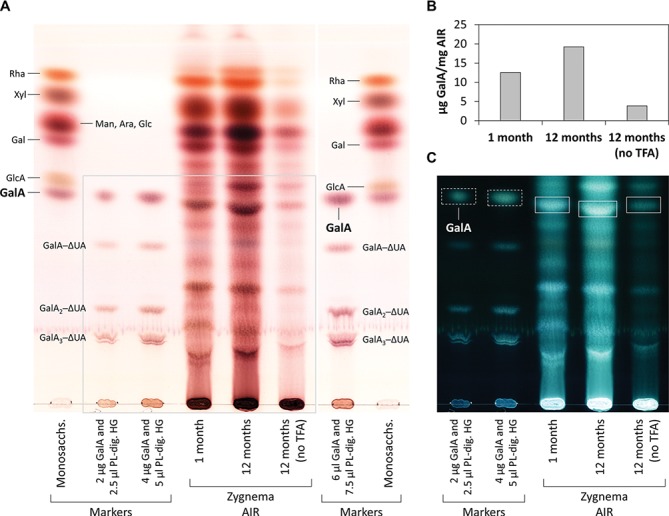
Monosaccharide and oligosaccharide composition of young and old *Zygnema* AIR. **(A)** Samples were TFA-hydrolyzed or left in water and hydrolysis products separated by thin-layer chromatography (TLC). TLC plate area shown in **(C)** is marked with a grey line. **(B)** Quantification of GalA in AIR samples treated or not treated with TFA. **(C)** Same plate as in **(A)**, but shown with inverted colors. TLC marker bands as in Figure 6. GalA bands in *Zygnema* AIR used for quantification (rectangles) and GalA marker bands (rectangles with dashed lines).

Digestion of commercial HG with PL produced the oligosaccharides GalA_1–3_–ΔUA but not the monosaccharide GalA, which we added additionally to our TLC marker mixtures (Figure 6A, second row from left and third and fourth rows from right). When incubated with PL, AIR of both young and old *Zygnema* filaments yielded a number of oligosaccharides and monosaccharides (Figure 6A). However, control groups lacking PL showed a similar oligosaccharide pattern upon TLC separation as PL containing samples (Figure 6A). Nevertheless, old filaments’ AIR consistently yielded more GalA_2_–ΔUA than young filaments; other oligosaccharide products diagnostic for PL digestion of HG (GalA–ΔUA, GalA_3_–ΔUA) were not found (Figure 6A). However, GalA, which indicates the presence of HG, was detected in all samples, and the amount was higher in old AIR (Figure 6A). To further validate that *Zygnema* cell walls contain HG releasable by enzymatic treatment, old *Zygnema* AIR was subjected to digestion by EPG, which cleaves the α-1,4-d-galactosiduronic linkages in HG. Separating EPG digestion products by TLC confirmed the release of GalA and an oligosaccharide running between the GalA and GalA─ΔUA marker band (Figure 6B), suggesting it to be GaA_2_. The higher amount of GalA in old samples as found in the PL digestion experiment was confirmed by TFA hydrolysis of additional *Zygnema* AIR (Figure 7A): GalA was ~50% higher than in young samples (Figure 7B). Control samples, where TFA was replaced by water, yielded only small amounts of monosaccharides and oligosaccharides (Figures 7A,C). Migration of TFA hydrolysis products was slightly retarded due to heavy TLC plate loading (especially in old AIR samples; Figure 7C).

## Discussion

The present study has shown that removing HG from the cell walls of the aero-terrestrial green alga *Zygnema* reduces its photosynthetic efficiency when exposed to water scarcity. The effect is the strongest in 12-month-old filaments, which exhibit a higher HG content than young filaments. This suggests that the pectin fraction in the cell wall helps algae to remain photosynthetically active in habitats with periods of low water availability. We thus conclude that the water holing capacities of HGs are crucial for survival in terrestrial habitats.

### HG Accumulation in Cell Walls of Old Filaments Might Be an Adaptation to Water Scarcity

Desiccating algal filaments with an intact HG matrix, i.e., without PL treatment, gave similar results as observed in previous desiccation studies on *Zygnema* (Kaplan et al., 2013; Pichrtová et al., 2014a,b; Herburger et al., 2015): (1) pre-akinete-rich old filaments maintained a higher photosynthetic performance during desiccation than young filaments and (2) full recovery of photosynthesis after rehydration only occurred in old filaments. Treatment of young filaments with PL slightly reduced their desiccation resistance, while the Y(II) reached 0 about 1 h earlier than in untreated filaments. This indicates that the HG matrix influences the water holding capacity of young filaments during desiccation. Furthermore, an intact HG matrix appears to be important for recovering photosynthesis upon rehydration: only untreated filaments and filaments which were allowed to recover their HG matrix restored their Y(II) partially after a 24 h rehydration period. Algae might prioritize the allocation of metabolic energy to the re-synthesis and incorporation of HG into the cell wall, and building HG-containing layers on top of the cell walls before the photosynthetic apparatus is repaired. In contrast to young filaments, HG removal in old filaments resulted in a drastic change of the desiccation kinetics: While untreated cells maintained a high Y(II) throughout the desiccation period (10 h), Y (II) and thus photosynthesis were inhibited in PL-treated filaments after ~9.5 h. Thus, PL-treated old filaments only exhibited a slightly higher desiccation resistance when compared with unt`reated young filaments, indicating that the age-dependent HG accumulation is important for coping with water scarcity. Cellular water loss results into dramatic morphological changes in *Zygnema* (Herburger et al., 2015; Lajos et al., 2016), where the longitudinal cell walls expand and show convolutions along the filament’s axis. In contrast, the cross cell walls remain their shape and stay connected to the protoplasts. Regular cell wall deformation patters are also considered to contribute to the desiccation tolerance of resurrection plants, where arabinose-rich components might mediate cell wall flexibility, allowing a controlled shrinkage during water loss (Moore et al., 2013). Although algae’s morphology after desiccation stress was not re-investigated in the present study, strong damage to the cell wall of PL-treated filaments can be excluded, because a recovering period allowed to restore the photosynthetic performance. This suggests that HG is not crucial for the cell wall’s structural integrity, even though it is a structural cell wall component (see below). Recovery of old filament’s HG matrix was accompanied by a gradual recovery of photosynthesis, resulting into restoration of the initial performance after 72 h. In contrast to young filaments, old filaments were able to restore their Y(II) partially, even when they were not allowed to recover after PL treatment. This indicates the acquaintance of further desiccation resistance mechanisms resulting from an age-dependent acclimation to desiccating conditions when grown on solid media (Rippin et al., 2017). For example, old filaments exhibit a lower osmotic potential inside cells (Kaplan et al., 2013) and a lower degree of vacuolization (Herburger et al., 2015), and the accumulation of lipid bodies might help maintaining the structural integrity of the protoplast during cellular water loss.

### HG Matrix Occurs Throughout Zygnema Cell Walls

Old *Zygnema* filaments are predominately comprised of pre-akinetes, which are considered to be crucial for long term population survival (Pichrtová et al., 2016a). As shown recently, the cell walls of pre-akinetes possess a higher pectin content than the walls of young vegetative cells, while the cellulose and hemicellulose contents are similar (Herburger et al., 2018). HG is the predominant polysaccharide in the pectin fraction of late diverged CGA (Sørensen et al., 2011), and correspondingly, we found GalA_2_─ΔUA oligosaccharides released by PL from *Zygnema* AIR. Surprisingly, high amounts of GalA_2_–ΔUA were also released in control samples (no PL) in a number of independent experiments; this might be caused by degradation of HG by β-elimination due to the relatively alkaline volatile buffer system used (pH 8; Renard and Thibault, 1996). Accordingly, acidic conditions (pH 4.5)—as used in the control samples in the EPG digestion experiment—did not yield any products, while adding EPG released GalA and (putatively) GalA_2_ from *Zygnema* AIR. TFA hydrolysis of Zygnema AIR also released GalA, which most likely resulted mainly from HG; although cell walls of *Zygnema* (Figure 7) and other CGA (Popper and Fry, 2003; O’Rourke et al., 2015) contain both GalA and rhamnose, only small amounts of them are considered to be a part of rhamnogalacturonan I (Sørensen et al., 2011). The release of both GalA_2_–ΔUA and GalA was higher in old filaments. Abundant occurrence of HG in *Zygnema* was further supported by strong binding of the mAb JIM5 (recognizing partially methyl- and unesterified HG) in both young and old *Zygnema* filaments. Epitopes were distributed homogeneously throughout the longitudinal cell walls. HG being a structural part of the algal cell wall—as observed in land plants—is supported by the observation that PL treatment increases the binding of Abs recognizing xyloglucan and mannan epitopes, suggesting that HG is tightly associated to cell wall hemicelluloses (Herburger et al., 2018). In the Zygnematophyceae *Netrium digitus*, JIM5 also gave a general cell wall staining and additionally stained the mucilage vesicles strongly, while the HG probe JIM7 (recognizing partially methyl-esterified HG) stained the growing zones of the cell center strongly (Eder and Lütz-Meindl, 2010). In *Micrasterias denticulata*, JIM5 and JIM7 mAbs gave labeling of the young primary cell wall but did not stain the secondary cell walls (Eder and Lütz-Meindl, 2008). JIM7 produced a stronger signal, while JIM5 labeling predominated in the nongrowing cell wall areas. Furthermore, a strong cross reactivity of both mAbs to the extracted mucilaginous fraction was observed (Eder and Lütz-Meindl, 2008). Also in *Desmidium swartzii*, JIM5 and JIM7 staining was observed in the developing septum (Andosch et al., 2015).

However, we did not observe JIM5 binding in the amorphous pectic sheath layer, which surrounds *Zygnema* filaments. Nevertheless, the sheaths are stained by ruthenium red, suggesting the presence of negatively charged compounds other than partially or unesterified HG. These sheaths might exhibit a chemical and functional similarity to the mucilage layers secreted by, for example, the unicellular CGA *Micrasterias*, which are rich in acidic polysaccharides (Menge, 1976; Brosch-Salomon et al., 1998; Eder et al., 2008). Pectic sheath layers were also found in *Zygnema irregulare* upon pre-akinete formation under desiccating conditions (Fuller, 2013). *Z. irregulare* belongs to the same major genus subclade as *Zygnema circumcarinatum* investigated in the present study; however, the pectic sheath layers stainable by ruthenium red observed in this study were considerably thinner and often did not cover the entire surface of filaments. These differences might be caused by different growth conditions, while *Zygnema irregulare* experienced continuous desiccation stress but *Zygnema circumcarinatum* did not. We found pectic sheath in both young and old filaments and—in addition to HG in the cell wall—these mucilaginous layers might contribute to desiccation resistance by binding water in close proximity to the cell surface. PL treatment removed the majority of HG epitopes recognizable by JIM5. A similar effect was observed in *Penium*, where PL treatment was allowed to remove the HG-rich lattice attached to the cell surface (Domozych et al., 2014). PL-treated *Zygnema* filaments, which were allowed to recover for 10 h, showed JIM5 binding in areas close to cross cell walls, while after 3 days, HG was detectable again throughout the longitudinal cell walls in both young and old filaments. This indicates that HG is initially secreted at the poles of individual *Zygnema* cells—likely in methyl-esterified from—and as the cells grow, HG might become de-methylesterified by PMEs. This is supported by the observation that JIM7 (recognizing partially methyl-esterified but not un-esterified HG) only binds to cell wall areas at cell poles, while JIM5, which can detect not only methyl but also unesterified HG, stains the cell wall from cell pole to cell pole. As shown by Domozych et al. (2014), the HG-rich lattice, which is part of the outer cell wall layer of *Penium* cells, is formed by secretion of highly methylesterified HG at an isthmus zone, which separates two semicells; during cell growth, PMEs remove methyl groups from HG, resulting into a HG lattice that is predominated by HG with a low DE distal from the isthmus (Domozych et al., 2014). The authors emphasize similarities between the algal lattice and the HG-rich middle lamella of land plants (localization, formation pattern, and relevance for cell adhesion), and this might also partially apply to *Zygnema* cell wall areas stainable by JIM5 and JIM7. The relatively fast re-formation of JIM5 epitopes upon PL treatment suggests that in addition to its function as a structural cell wall component, HG might be also secreted to the outer cell wall surface, where it increases filaments water holding capacity and adhesion to the substrate and neighboring cells/filaments. The ability of *Zygnema* to form dense mats improves the water holding capacity at the population level and protects individual filaments from harmful irradiation (Holzinger and Karsten, 2013). Adhesive compounds such as HG and AGPs (as recently demonstrated in *Zygnema* by Palacio-López et al., 2019, this issue) on the cell surface might help algae to form these mats.

## Conclusion

*Zygnema*, the species-richest genus of the algal ancestor group of all land plants (Zygnematophyceae), shows an age-depend accumulation of HG in the cell wall. Older filaments exhibit a higher resistance against desiccation stress, as previously shown by different approaches (Kaplan et al., 2013; Herburger et al., 2015; Rippin et al., 2017). We suggest that a higher HG content of the ECM allows the filaments to bind more water, which gives them their typical slimy appearance and delays a harmful cellular water loss. It is likely that this adaptation supported algae in colonizing land and helps recent *Zygnema* populations to survive in harsh aero-terrestrial habitats throughout the world. With this increased water holding capacity, a longer productivity range is expected, leading to massive amounts of biomass created even under partially desiccating scenarios (Holzinger and Pichrtová, 2016).

## Author Contributions

KH and AH planned and designed the study during KH’s PhD appointment at the University of Innsbruck. KH performed most of the experiments and analyzed the data. AH conducted immuno-TEM and prepared AIR samples. AX performed the HG quantification assays. KH prepared the figures and wrote the manuscript draft. All authors edited the final manuscript.

### Conflict of Interest Statement

The authors declare that the research was conducted in the absence of any commercial or financial relationships that could be construed as a potential conflict of interest.
